# Heat, Health and the Himalayas: Tackling Health System Impacts of Global Climate Change

**DOI:** 10.31729/jnma.v64i293.9285

**Published:** 2026-01-31

**Authors:** Aishana Joshi

**Affiliations:** 1 Editor, Journal of Nepal Medical Association, Pradashani Marg, Kathmandu, Nepal.

Climate change presents a fundamental threat to human health and well-being. The global climatic shifts have contributed to extreme weather events, worsening air quality, spread of infectious and vector-borne diseases and threats to food and water security, largely affecting global health and imposing insurmountable strain on health systems. Among the most immediate and visible impacts of climate change is the rising heat which poses significant risks to human health and unprecedented challenges for health systems worldwide, intensifying existing gaps and inequities in health care.^1,2^ In recent years, global warming has led to a rise of 1.1°C in mean global temperature since the Industrial Revolution, with projected increase of 2.5 to 2.9°C by the end of the century if drastic reductions in greenhouse gas emissions are not achieved.^3^ The Himalayan region, traditionally associated with cool mountain climates are warming at rates exceeding the global average resulting in ecological fragility and increased population vulnerability.^4,5^ Global warming has led to the emergence of heat waves in temperate and cooler climate regions, overwhelming indigenous populations and health systems unaccustomed to extreme heat, increasing the risk of heat-related morbidity and mortality. Vector-borne diseases such as dengue, once largely confined to the lowland areas are now being reported at elevations above 2,000 meters; regions previously considered unsuitable for mosquito survival.^6,7^ This geographic expansion reflects altered vector ecology and changing epidemiology of climate-sensitive diseases in high altitude and mountainous regions, attributed to rising temperatures.^8^ In geographically remote Himalayan settings, these challenges are further compounded by transportation barriers, limited health care infrastructure and resource constraints straining the health systems.

Extreme heat driven by global climate change is not merely an environmental concern but an escalating threat to health systems. In Nepal and across many low- and middle-income countries (LMIC), inadequate health infrastructure leaves facilities poorly equipped to cope with rising temperatures.^9^ Beyond health facilities, the vast majority of public housings, academic institutions, offices and factories operate without optimal temperature control. The lack of air conditioning, insufficient ventilation, inadequate building insulation and intermittent electricity supply continue to widen the “cooling gap” particularly in low resource settings. Extreme heat disrupts infection control and cold chain systems, compromises health workforce performance and undermines the quality of care.^10^ Prolonged exposure to high temperatures leads to heat stress, exhaustion and heat stroke impairing both physical and cognitive functioning.^11^ Rising temperatures also increase demand for emergency services, outpatient care and hospital admissions due to surges in heat related illnesses, exacerbation of cardiovascular and respiratory diseases, acute kidney injury, poor mental health and heightened risks of infectious and vector-borne disease outbreaks.^12-14^ Despite these impacts, integration of climate data into routine health planning remains limited. Health information systems often lack standardized indicators for heat-related health impacts limiting the ability to quantify disease burden, identify high-risk populations and design effective interventions.

Health system strengthening is imperative and central to effective climate action; a vital investment in climate adaptation, health equity and sustainable development. Health systems must be operationally prepared to address the multifaceted challenges posed by climate change. Frameworks from the World Health Organization (WHO) and the Intergovernmental Panel on Climate Change (IPCC) emphasize the importance of climate-informed health surveillance in enhancing health system resilience to extreme heat.^3,15^ Key priorities include integrating climate risk into health planning, upgrading infrastructure for thermal comfort and energy efficiency, designing early warning systems, protecting health workforce through training and occupational safeguards and strengthening heat-health surveillance. Fostering robust research capacity is critical for generating context-specific evidence to inform policy and guide locally appropriate interventions in climate-vulnerable communities. Intersectoral collaboration linking health systems with urban planning, energy, water and disaster risk reduction is essential in mitigating risks, enhancing adaptive capacity and building community resilience. Addressing these gaps is critical to anticipate heat-related impacts, guide timely interventions and enhance health system preparedness. This is a call to action for collaboration to co-create evidence-based and equity-focused solutions that place health systems at the heart of climate action.
